# The Story of My Life

**Published:** 1885-06

**Authors:** 


**The Story of My Life.** By J. Marion Sims, M. D., LL. D. Edited by his son, H. Marion Sims, M.D. New York: D. Appleton and Company, 1884.

The history of self-made men, who rise from obscurity to fame by dint of their own personal strength of character and will, is always attractive reading to an American; yet there is, ordinarily speaking, no novelty in such a record. The bookshelves of our libraries are full of "Lives" of men who have risen to the high pinnacles of renown from the humblest walks of life. These are not uncommon events in the history of the mart, the forum, or even the pulpit, but in medicine such an occurrence happens much more rarely. The number who attain prominence in our profession is so large that only a few can be truly great, and they generally have become so by reason of some especial advantage, either of birth, locality or favor.

In the story of Dr. Sims' life, we read the triumphs of genius over the most adverse environments; and its charm is not lessened by the simplicity of the narrative. Born in South Carolina in 1813, his paternal ancestors were of English, and his maternal of Scotch-Irish origin; and though without the advantages of early education, his parents were of sturdy character and noted for those strong qualities of head and heart which distinguished the men and women of the early part of the present century. His youthful and college days were unmarked by special incident, though full of interesting experiences. But from the time of his graduation in medicine, in 1835, to his final establishment of the Women's Hospital in New York, in 1857, his life was filled with episodes and incidents, many of which possess thrilling interest. His graphic description of the investigation of, operation for, and final cure of vesico-vaginal fistula, continued during a period of four years, when, after repeated failures, he was finally successful in a case upon which he operated thirty times, is already embalmed in the history of American Medicine. Silver wire in surgery had its birth during the series of investigations of this terrible, and hitherto uncured, accident of parturition; and his ultimate success in the operation was ascribed to his invention of the silver suture.

There is no more pathetic picture in medical literature than the devotion of the seven or eight young colored women, all suffering with vesical fistules, whom he succeeded in inspiring with a confidence which clung to him, amid trials and defeats when all others deserted him and who submitted to repeated operations, so painful that none but a woman could endure. His failing health led to his subsequent removal to New York, where, after a few years spent in struggling with ill-health and poverty on the one hand and bitter opposition on the other, he finally succeeded in establishing the Women's Hospital, the first institution devoted exclusively to the treatment of the surgical diseases of women, which the world had yet seen. In this enterprise he encountered the strongest opposition of the greatest men in the profession; for he belonged to no clique or party. But having enlisted the sympathy and support of a few good, true and philanthropic women, who gave substantial aid and comfort to the scheme, he overwhelmed his opponents so completely that many of them were subsequently among his ablest coadjutors in perfecting plans for the hospital and establishing it upon an enduring basis.

It was in this hospital that American gynecology obtained its first impetus, and it was here stamped with its birth-mark by the originality and brilliancy of its first master. The men who Dr. Sims here taught have since become masters themselves, and the beneficial influences of his and their labors have made a lasting impression in two hemispheres.

The domestic life of Dr. Sims presents a most charming picture of conjugal devotion, virtuous simplicity and filial and parental affection. The sweetheart of his youth was no less the sweetheart of his maturer years, and to her influence, advice and judgment was due in a large measure his great success in life, which he gracefully acknowledges in a most delightful manner.

For several years he was the victim of that almost universally fatal malady of a warm climate, chronic diarrhœa, during which period he came nigh unto death several times; all his food during all these long years was prepared by the loving hands of his faithful, devoted and accomplished wife.

This book cannot fail to exert a good influence upon both the professional and lay readers, and especially do we hope it will find its way into the hands of young physicians. To them its lessons of patience, courage, perseverance, industry, honor and virtue cannot fail to be of value, when, in their early years of professional life, they so often experience the heart sickness of hope deferred. The life of Dr. Sims is an example worthy of any man's emulation. He was a man who wrung victory from the jaws of defeat as often as ever any man did. Poverty, ill-health and opposition were no hindrance to him—he triumphed over them all, singly and combined, even when arrayed in all the panoply of fierce and deadly conflict.

The defects in style of composition, which almost necessarily creep into such a purely personal story, are easily overlooked or forgotten in the absorbing interest of the narrative, and we advise our readers to possess themselves of this life of the foremost medical man of his time.

